# Discovery and biodegradation characterization of polyethylene by *Metabacillus niabensis*

**DOI:** 10.3389/fmicb.2025.1693690

**Published:** 2025-12-03

**Authors:** Raj Kumar Sardar

**Affiliations:** 1 CSIR-Central Salt and Marine Chemicals Research Institute (CSIR-CSMCRI), Council of Scientific and Industrial Research (CSIR), Bhavnagar, Gujarat, India; 2 Academy of Scientific and Innovative Research (AcSIR), Ghaziabad, India

**Keywords:** *Metabacillus niabensis*, SEM, FTIR, GC–MS, dehydrogenase, peroxidase, polyethylene

## Abstract

Polyethylene (PE), a type of plastic, is the primary contributor to persistent and prolonged environmental contamination. Plastic biodegradation is considered a promising approach to addressing current environmental issues. In this study, 300 marine isolates were evaluated for their ability to biodegrade PE. Based on total cellular fatty acid profiling and 16S rRNA gene sequence homology, RS120 was identified as *Metabacillus niabensis* (*M. niabensis*) among positively tested marine strains. Furthermore, early bacterial attachment to the PE surface was observed during the biotreatment. Then, after, chemical structural changes before and after the biodegradation were shown by the disappearance of the larger hydrocarbon tetra-tetracontane (C_44_H_20_) and the emergence of the smaller hydrocarbon heptadecane (C_17_H_36_), tetracosane, benzene, 1,1′-(cyclobutanediayl) (C_16_H_16_), and 2-tert-butyl-4-6-bis (3-5-di-tert-butyl). After that, Fourier Transform Infrared Spectroscopy (FTIR) spectra designated two new, shouldered peaks at 2590 cm^−1^ and 2,610 cm^−1^ along with a longer, sharper, and pointed peak at 2361 cm^−1^ in weathered PE, which substantiates significant alteration in chemical structure. Moreover, significant variation in thermal stability and crystallinity of plastic was also investigated. According to the enzymatic studies, bacterial peroxidase and dehydrogenase activities during treatment were higher at 30 days of treatment. In the meantime, the weight loss of the film was regularly monitored, and within 30 days of co-incubation with RS120, about 3.3% of its initial weight had been lost. These findings showed that the strain partially degrades the PE film when used as the sole carbon source. The results revealed that *M. niabensis* is reported here for the first time as an efficient PE degrader.

## 1 Introduction

In many applications, plastic materials have been preferred over metal, wood, and leather products in recent decades. Many facets extensively utilize it due to its durability and stability (Andrady and Neal, 2009; Ganesh et al., 2020). A study by Ritchie and Roser (2018) predicts that by 2034, annual plastic production will exceed 320 million tons. The amount of plastic produced worldwide has increased from 2 million tons in 1950 to 450 million tons in 2019, and it is projected to reach 507 to 537 million tons by 2025 (Bahl et al., 2020). India has also become a hotspot for plastic pollution, largely due to its expanding economy, which produces approximately 9.4 million tons of plastic annually (Joshi et al., 2025). Ironically, they constituted a thoughtful ecological issue due to their xenobiotic and resistant characteristics. Plastic garbage can now be disposed of in three primary ways: recycling, incineration, or landfill disposal (Ignatyev et al., 2014). In addition to releasing hazardous chemicals like hydrogen chloride and hydrogen cyanide, which are harmful to both animals and the environment, burning plastic also releases poisonous gases such as furans and dioxins (Shahnawaz et al., 2016). Between 60 and 80% of all marine garbage in the marine ecosystem is composed of plastic (Derraik, 2002). The water is disposed of, along with almost one million tons of plastic, per year. Conversely, landfilling plastic waste has a detrimental effect on the fertility of the surrounding area and the quality of life for the local population (North and Halden, 2013). Furthermore, a lot of birds are killed by strangling or ingesting plastic debris from rivers and city drainage systems that get into the ocean, even though plastic products have been found in human tissue such as brain, fat, nasal secretions, urine, and serum, indicating widespread human exposure to them (Harray et al., 2024).

Polymer degradation is caused by a combination of chemical, biological, and physical processes. According to Ojha et al. (2017), microbial-mediated degradation is an environmentally benign method that involves reducing the average molecular weight by microorganisms, backbone chain breaking, changes in surface character, and a loss of mechanical strength. Consequently, energy is generated from the products of microbial breakdown of plastics (Peixoto et al., 2017). In biodegradation, enzymes play a crucial role and belong to two enzyme classes: intracellular and extracellular depolymerases (Tournier et al., 2023). Large complex polymers are first broken down into smaller molecules, such as monomers, dimers, and oligomers, and then they are mineralized. These are also too small to pass through the exterior, semi-permeable gate and can be metabolized after being taken up and biodegraded within the microbial cell. After that, the fatty acid undergoes oxidation within the cell. The process is called depolymerization. When CO₂, H₂O, or CH₄ are the end products, the degradation is referred to as mineralization (Lv et al., 2024).

Enzymes and genes that break down resistant pollutants, such as plastic waste, are found in marine microorganisms. The biodegradation of plastics facilitated by microorganisms involves microbial attachment, in which a hydrophobic surface becomes more hydrophilic, leading to early physical and chemical breakdown through the introduction of hydrophilic groups. The microbe begins to develop by consuming the degraded PE as a source of carbon. The ocean contains a vast array of microorganisms that may contribute to the decomposition of synthetic plastic materials. Among the microorganisms implicated in the biodegradation of polyethylene (PE) are fungi, bacteria, and actinomycetes. The marine-sourced *Pseudomonas, Moraxella, Micrococcus, Streptococcus,* and *Staphylococcus* were identified as plastic degraders, with degradation rates ranging from 2.19 to 20.54% (Chandra and Singh, 2020). Furthermore, numerous studies on marine bacteria have demonstrated that they can break down plastic; some of these bacteria include *Bacillus cereus* and *Bacillus sphericus* (Sudhakar et al., 2008). Furthermore, *Bravibacillus borstelensis* and *Rhodococcus ruber* have also been shown in studies to be able to colonize and subsequently break down PE (Sivan et al., 2006; Hadad et al., 2005). However, degradation can be achieved by enhancing the biodegradability of plastics by combining them with cellulose or starch and pro-oxidants to facilitate their easy breakdown and, secondly, by enhancing microorganisms that efficiently break down polymers.

The genus *Metabacillus* is a reclassified group of bacilli. To better reflect the variety within the original *Bacillus* group, Patel and Gupta (2020) proposed the *Metabacillus* as one among six new genera. There were numerous *Bacillus* species, including previously reported species, that were moved to the new genus. *Metabacillus fastidiosus* was the first isolate of the *Metabacillus* genus. The original name of this species was *Bacillus fastidiosus* (Dooren and de Jong, 1929). Following the 2021 reclassification of the genus *Metabacillus*, the *Bacillus niabensis* 4T19T was also renamed as *M. niabensis*, *which* was originally isolated from cotton-waste compost used in mushroom growth (Kwon et al., 2007). A variety of habitats, including soil, hypersaline lakes, and marine coastal areas, were used to isolate the *Metabacillus* species. The cellular proteins that distinguish *M. niabensis* from its other species include the flagellar M-ring protein, the 50S ribosomal protein, the ATP phosphoribosyltransferase regulatory subunit, and others (Patel and Gupta, 2020). Some species can withstand moderate levels of salt (0–5%), and they can be grown at temperatures between 4 and 45 °C, with 25–37 °C being the ideal/optimum range for growth. In summary, *M. niabensis* is an aerobic, motile, Gram-negative bacterium with a genomic size of 4,987,608 bp and 4,969 recognized genes (Kangale et al., 2021). Bacteria from marine sources typically exhibit shorter-chain fatty acids to counteract the freezing effect of cold on long-chain fatty acids, enabling them to survive in harsh climates. Short-chain fatty acids preserve the physiological fluidity of the outer membrane (Sardar et al., 2015).

Since only a tiny fraction of the PE will be absorbed into humus, bacterial cellular biomass, and other ordinary products, the complete bio-deterioration and biodegradation of plastics is not yet feasible (Ghatge et al., 2020). Changes in physical properties and chemical structure of the shredded polymers were carried out using scanning electron microscopy (SEM), gas chromatography mass spectroscopy (GC–MS), Fourier transform infrared (FTIR) spectroscopy, X-ray diffraction (XRD), differential scanning colorimetry (DSC), and weight loss recorded in order to uncover the possible degradation mechanism of the bacteria that break down PE. Additionally, the activity of enzymes in the biological degradation of PE polymers after bacterial treatment was hypothesized.

The goal of the current investigation was to identify a new, potent strain of marine isolate capable of degrading PE. The further aim is also to assess and characterize the degree of PE degradation using the discovered marine isolate. Finally, these studies tested the ability of culturable marine bacteria to degrade PE. After identifying the putative marine bacterial isolate, their 30 day PE-degrading capacity was evaluated. *M. niabensis* is the first species of the genus *Metabacillus* to be investigated and shows the ability to degrade PE.

## 2 Methods

### 2.1 Polyethylene

The polymer used as the carbon source in this study was a medium-density polyethylene (MDPE) plastic film, 10 μm (0.010 mm) thick, purchased commercially from Good Fellow Cambridge Ltd. in Huntingdon, England, to serve as the foundational material. The MDPE is a moderately branched polymer with a molecular weight comparable to that of LDPE and HDPE plastics. MDPE’s density ranges from 0.926 to 0.940 g/cm^3^. Following a 1 mm × 1 mm incision, the polymer films were weighed, disinfected with 70% ethanol, and dried.

### 2.2 Marine bacteria

The bacterial strain RS120 used in the PE plastic biodegradation study was among the marine bacteria (300 marine bacteria) used for screening for biodegradation. These bacterial strains were received from the laboratory culture collection repository at the Applied Phycology and Biotechnology Division, CSIR-CSMCRI, Bhavnagar, which were earlier collected from salt water from Sikka, Gujarat, on the Arabian Sea coast (latitude N 22° 25′98.0″ and longitude E 69° 49′3.85″) in India. Following a 24–48-h incubation at 30 °C, these isolates were revived on Zobell Marine Agar medium (Himedia). After isolating and transferring pure bacterial colonies into Zobell Marine Broth, they were allowed to grow and then purified. These cultures were then stored at −80 °C in 10% glycerol.

### 2.3 Preliminary screening of PE degrading marine bacteria

Before the biodegradation experiment, tiny 1 cm x 1 cm pieces of PE film were cut, cleaned, weighed, and sterilized. Before use in any experiment, these tiny strips were sterilized by immersing them in 70% alcohol, then exposed to UV light and dried in a sterile condition. Then after this, sterilized PE films (weight 100 mg) were added to 20 mL of carbon-deficient Bushnell Hass (BH) minimum broth media supplemented with 3% NaCl (Eriksson et al., 2000). In additional culture tubes, 20 μL of 2, 3, 5-triphenyl tetrazolium chloride (TTC) indicator and 1% fresh bacterial inoculum of the RS120 strain were added to assess the survival of bacteria in BH media devoid of carbon content. The color of the media altered over the incubation period, according to Tan et al. (2021), suggesting that bacteria were using the carbon from PE for growth. Similarly, one treatment served as the control, with no bacterial inoculation, and three duplicates of each treatment were used to produce the blanks. Sets of every tube were incubated for 30 days at 180 rpm and 30 °C in an aseptic environment. Every 7 days, the plastic films were removed in triplicate from the culture vessels and subjected to a range of analytical measurements to illustrate bacterial attachment and PE film degradation.

### 2.4 Identification of selected performant strain

#### 2.4.1 Total cellular fatty acids profiling

Whole-cell fatty acid contents of strain RS120 were transformed into methyl ester and analyzed by gas chromatography (GC). For the Fatty Acid Methyl Ester (FAME) study, the sample was prepared in five steps, viz., harvesting, saponification, methylation, extraction, and base washing, following the MIDI FAME protocol. The prepared methyl ester extract was analyzed using a GC 6850 series (Agilent Technology, Hewlett-Packard, Wilmington, DE, USA), which detected straight-chain saturated fatty acids extending in length from nine to twenty carbons, as well as five hydroxyl acids, along with a calibration standard (Supleco). The GC 6850 series was equipped with a 30 m × 0.25 mm × 0.25 mm fused silica capillary column, utilizing very high-purity hydrogen as the carrier gas, and a flame-ionization detector (FID). The initial oven temperature was 170 °C, increased by 5 °C min^−1^ to 260 °C, then by 30 °C min^−1^ and held constant at 300 °C for 2 min. Helium gas was applied as a carrier at a flow rate of 1.0 mL min^−1^ (Sasser, 2006; Veum et al., 2021). Retention durations for fatty acids were compared to a bacterial fatty-acid reference mix (bacterial acid methyl ester mix CP, 4–7,080; Supleco, Inc., Bellafonte, PA). Agilent Technology uses the RTBS6 library database for matching the FA.

#### 2.4.2 Molecular identification and phylogenetic analysis

Marine strain RS120 was selected for its high potential to degrade PE compared to other screened microorganisms that grow on PE and underwent genotypic identification by 16S rRNA gene sequencing. The total genomic deoxyribonucleic acid of strain RS120 was extracted using the conventional phenol-chloroform technique (Weisburg et al., 1991). For amplification of the 16S rRNA gene, the universal primers 27F (50-AGAGTTTGATCCTGGCTCAG-30) and 1492R (50-CTACGGCTACCTTGTTACGA-30) were utilized (Galkiewicz and Kellogg, 2008). Each primer (10 pmol) was added to a 50 μL reaction mixture together with dNTPs (400 μM), Taq polymerase (2.5 U), MgCl₂ (1.5 mM), DNA template (40–50 ng), and 10X PCR buffer (5 μL). Amplification of the 16S rRNA gene was conducted using the following PCR program: initial denaturation at 94 °C for 8 min; 30 cycles of 94 °C for 1 min, 58 °C for 1 min, and 72 °C for 2 min; and a closing extension at 72 °C for 10 min. Afterwards, the amplified product was purified using the PCR Purification Kit (Qiagen), and sequencing was facilitated by M/S Microgen Inc., Seoul, South Korea. Neighbor-joining (NJ), maximum-likelihood (ML), and maximum-parsimony (MP) approaches were employed to construct phylogenetic trees based on unambiguously matched sequences (Sardar, 2025) using MEGA version 11 software (Tamura et al., 2021). For each of the three techniques, 1000 bootstrap trials were carried out to assess the robustness of tree topologies.

### 2.5 Microbial colonization on PE film

SEM images visualized the initial adherence of strain RS120 to the PE surface during incubation. PE films were prepared for SEM imaging using the method illustrated by Joshi et al. (2022). Films were removed from broth, and attached particles and precipitates were achieved using phosphate buffer. Then, the films were rinsed lightly, treated with 2% glutaraldehyde, and dehydrated three times: once in 50% ethanol for 30 min, once in 70% ethanol overnight, and once in 100% ethanol for 30 min. After the samples were fixed, vacuum-dried, and coated with gold, a field emission scanning electron microscope (JSM-7100 F, Jeol Ltd., USA) was used to analyze them.

### 2.6 Characterization of PE-degradation

#### 2.6.1 GC–MS analysis

The culture filtrate (untreated and *M. niabensis*-treated) was removed after 30 days of treatment using diethyl ether solvent, following the extraction procedure. The cell-free supernatant was dissolved in diethyl ether for the extraction of the residual material with a 1:1 (v/v) ratio. With periodic shaking, this mixture was maintained at 100 °C in the water bath for six hours. During the incubation period, the mixture was removed from the water bath after 3 h, cooled to room temperature, and then returned to the water bath for an additional 3 h. The next step involved vortexing the content for 2 min and then holding it static for 10 min to allow the two phases to disperse. Using a glass Pasteur pipette, the organic phase was extracted from the bottom layer and analyzed by GC–MS (Shimadzu QP2020, Japan) to determine the breakdown products of PE. An AOC-20i autoinjector was used to inject 1 μL of the sample. At a pace of 6 °C per minute, the oven’s temperature rose from 50 °C to 280 °C. At a threshold of 1,000, the ion source was kept at 200 °C. A scan period of 0.30 s was established, spanning 50 to 500 m/z (Liu et al., 2022).

#### 2.6.2 FTIR analysis of PE films

Alterations in the PE structure were investigated using Fourier Transform Infrared (FTIR) analysis (PerkinElmer, Spectrum EX) during the ongoing bacterial incubation. After a month of incubation with bacterial treatment, both treated and untreated PE film samples were processed using the method described by Joshi et al. (2022). Dried and clean PE films were used for analysis by spectroscopy from the 4,000–400 cm^−1^ frequency band, which was employed with a 1 cm^−1^ resolution (Albertsson and Karlsson, 1990).

#### 2.6.3 XRD and DSC analysis

The X-ray diffractometer (Philips X’pert MPD, The Netherlands) was used to examine the crystalline structure of both treated and untreated PE. The *θ*/θ range at ambient temperature was 2° to 60°. With a 240 mm goniometer radius and a receiving slit size of 0.1 mm, the irradiation length and specimen length were both 10 mm. The focus and divergence slits were separated by 100 mm. Utilizing an h/h geometry with Cu Kα (k = 1.5406 Å) radiation, the system is equipped with an Ultima IV (Tokyo, Japan) goniometer. The operational parameters were I = 30 mA and U = 40 kV. Cleansed and dried PE thin film was placed on quartz substrata, and continuous measurements were made at d-spacings according to the diffracted X-ray at each θ value, with a step time of 1 s (Wu et al., 2023). Furthermore, the percentage crystallinity of both sets of PE film was calculated by using OriginPro 8.5 and the following formula to process the peaks at 2θ in the XRD data.


$$\text{Percent crystallinity}=\frac{\mathrm{Sum}\;\text{of crystalline peak area}}{\text{Total peak area}}\times 100$$

Thermal analysis of PE films was performed in nitrogen conditions (N₂, 20.0 mL/min/N₂, 70.0 mL/min) using a differential scanning calorimetry (DSC) machine (NETZSCH DSC 204F1 Phoenix 240–12-0239-L, Germany). Around 5.0 mg of plastic was weighed into an aluminum crucible pan covered with a pierced lid and heated from 24 °C to 250 °C (10.0 K/min). PE samples were cooled to 30 °C and then heated to 180 °C, 220 °C, or 300 °C, respectively (heating/cooling rates were 10 °C/min). Three observations, including glass transition temperature (T_g_), melting point (T_m_), and cold crystallization temperature (T_c_), were obtained from the DSC thermograms (Beltrán-Sanahuja et al., 2021).

#### 2.6.4 Determination of weight loss

The biodegradation of PE was assessed using a weight-loss assay. Every 7 days, the PE films treated with and without strain RS120 were removed from the culture tubes and weighed on a weighing balance with a readability of 0.1 mg (Shimadzu Analytical Pvt. Ltd., India). After three Milli-Q water washes, the plastic films were soaked in a 2% (w/v) SDS solution for 2 h. After the films were dried for the whole night at 50 °C in an oven, the molecular mass loss was computed as follows: Weight of treated PE minus weight of untreated PE (blank) equals total mass reduction (Sardar, 2025).


$$\text{Weight loss}\;(\%)=\frac{\left(\text{Initial weight})-(\text{Final weight}\right)}{\left(\text{Initial weight}\right)}\times 100$$

#### 2.6.5 Peroxidase and dehydrogenase activity

Since microbial enzymes play a role in plastic biodegradation, peroxidase assays were conducted using cell-free supernatants. At 7, 14, 21, and 30 days of treatment, culture supernatant was collected by centrifuging broth aliquots containing films at 5000 rpm for 5 min at room temperature. To test peroxidase activity, a mixture of 1 mL of crude enzyme, 4.0 mM hydrogen peroxide, 0.1 mM potassium phosphate buffer (pH 7.0), and 1.0 mM levodopa was used as the substrate (Zhao et al., 2004).

To conduct a dehydrogenase assay, a mixture consisting of crude cell lysate (150 μL), 1% triphenyl tetrazolium chloride (50 μL), and phosphate buffer (300 μL) was prepared and incubated for 15 min at 37 °C, then 500 μL of icy methanol was added to stop the reaction. The color shift was measured and noted in a spectrophotometer at 470 nm for peroxidase and 480 nm for dehydrogenase. The quantity of enzyme required to elevate 1.0 absorbance units per minute is equivalent to one unit of enzyme activity (Mayer and Arnold, 2002).

### 2.7 Statistical analysis

The experiment included five replications. Statistical analysis was performed using SigmaPlot 12. All values were presented as the mean ± SD. Differences between groups that were statistically significant (*p* < 0.05) were found using Duncan’s test and one-way analysis of variance (ANOVA).

## 3 Results

### 3.1 Screening of PE-degrading bacteria

Biological degradation of the PE film was anticipated by the marine bacterial strain RS120, as indicated by the TTC reduction assay. The colorless TTC turns into the red insoluble triphenyl formazan (TPF) when bacteria use the released carbon from the PE sheet as their sole energy source for growth in the BH Media (Supplementary Figure S1). The electron transport chain drives this activity. As indicated by the tube’s screening results, the capable strain RS 120 broth containing (tube A) turned red (Supplementary Figure S1) under the PE-supplemented condition, indicating that it was the only carbon source. While tube C does not exhibit redness when PE is absent. As shown in the tube (B), the absence of redness indicates that no microorganisms were present during the experiment, which verifies the lack of contamination. Therefore, during the screening process, the tested positive strain grew only when using free carbon elements released by PE films in BH broth, which turned the broth red, and was chosen as a bacterium that degrades plastic.

### 3.2 Identification of PE-degrading bacteria

#### 3.2.1 Total cellular fatty acids profiling

Whole cell fatty acid profiling of the PE-degrading strain RS120 exhibited the majorly branched fatty acids C15:0 ante-iso (51.55%), C15:0 iso (13.77%), and C17:0 ante-iso (8.74%), accompanied by some straight-chain C16:0 (4.69%) and C14:0 (1.20%), which are mentioned in Table 1. Their corresponding GC–MS peaks are also reflected in Supplementary Figure S2. In the hostile oceanic environment, the abundance of branched fatty acids and shorter chains in marine bacteria helps mitigate the environmental stress caused by temperature, pH, and salt concentrations.

**Table 1:** GC–MS: total cellular fatty acid profile of *M. niabensis* strain RS120 using method: RTSBA6.

Sl. No.	Retention time	Peak name /fatty acids	Percentage (%)
1.	1.8143	13:0 iso	0.61
2.	1.8393	13:0 anteiso	0.48
3.	2.0974	14:0 iso	5.31
5.	2.2057	14:0	1.20
6.	2.4009	15:0 iso	13.77
7.	2.4298	15:0 anteiso	51.55
8.	2.5152	15:0	–
9	2.6473	16:1 *w*7c alcohol	1.26
10.	2.7165	16:0 iso	5.76
11.	2.7647	16:1 w11c	2.13
12.	2.8335	16:0	4.69
13.	2.9669	17:1 iso *w*10c	0.69
14.	3.0366	17:0 iso	2.37
15.	3.0675	17:0 anteiso	8.74
16.	3.1540	17:0	0.62

#### 3.2.2 16S rRNA gene and phylogenetic analysis

**Figure 1:**
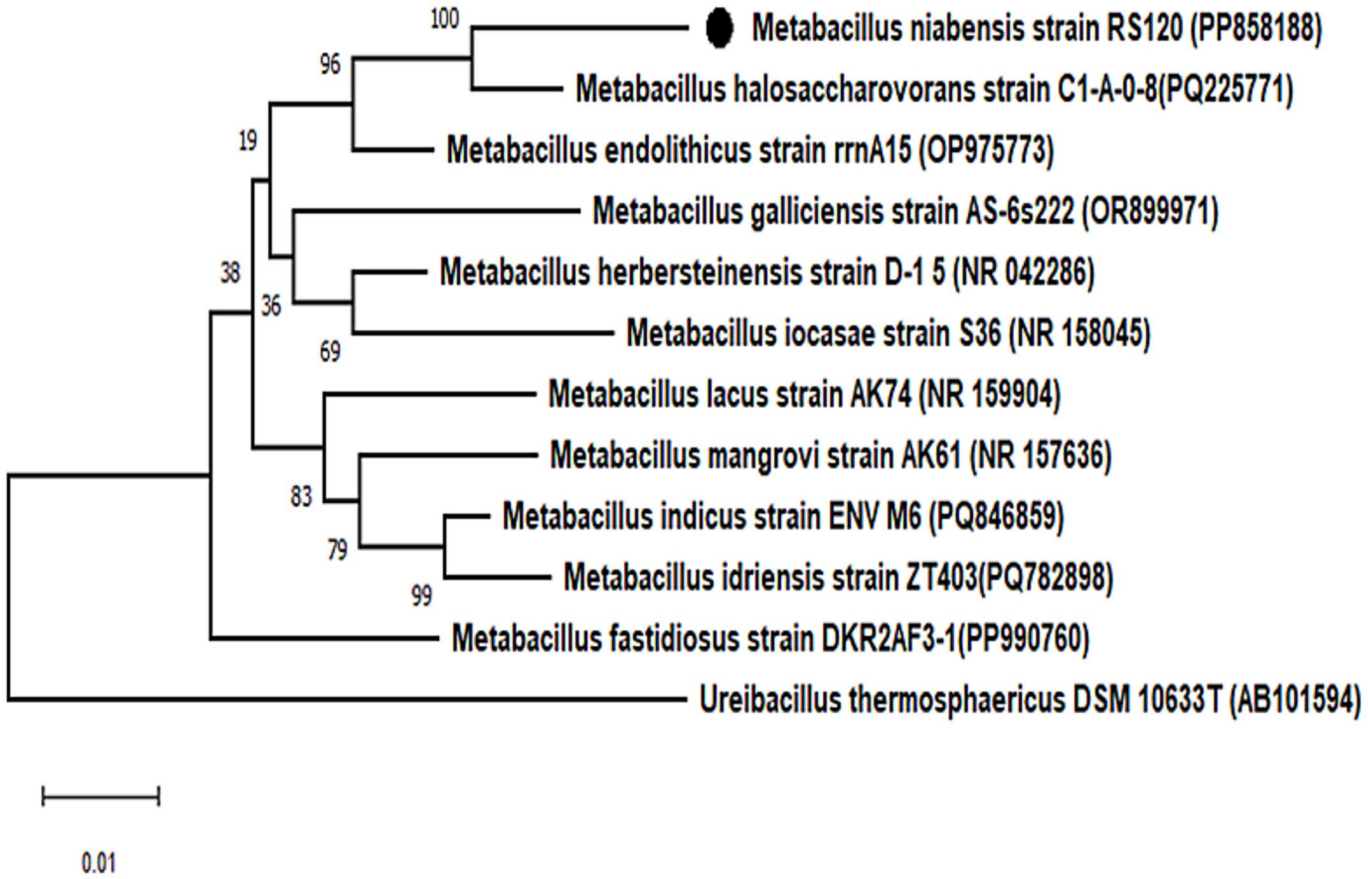
Phylogenetic analysis of bacterial strains. Phylogenetic tree based on 16S rRNA gene sequence showing the relationship between the isolated bacterial strain *Metabacillus niabensis* RS120 and other relatives within the genus. Bootstrap values greater than 60% are shown on branch nodes, which are based on 1,000 replicates. *Ureibacillus thermosphaericus* DSM 10633^T^, accession no. AB101594 served as the outgroup. Evolutionary analyses were conducted in MEGA 11.

A performant strain was identified and confirmed following completion of the BLAST study of the 16S rRNA gene. The organism was identified as a gram-positive *M. niabensis* strain 120 with a 99% homology match. Furthermore, the partial gene sequence of 16S rRNA (1,370 nucleotides) was submitted to the National Center for Biotechnology Information (NCBI) database, and the GenBank accession number is PP858188. According to a phylogenetic study with the NJ, ML, and MP algorithms, strain RS120 and *M. niabensis* (AY998119) formed a phylogenetic lineage within the genus *Bacillus*, as shown in Figure 1. The *M. niabensis* strain 120 showed taxonomically very close with the moderately halophilic bacterium *M. halosaccharovorans*, as depicted in phylogenetic analysis Figure 1, which was isolated from a hypersaline lake. The GenBank databases provided the reference sequences used for strain comparison. According to FAME and 16S rDNA sequence analysis, the isolate is native to the marine environment, consistent with other investigations (Romano et al., 2020).

#### 3.2.3 Microbial adherence using SEM

**Figure 2:**
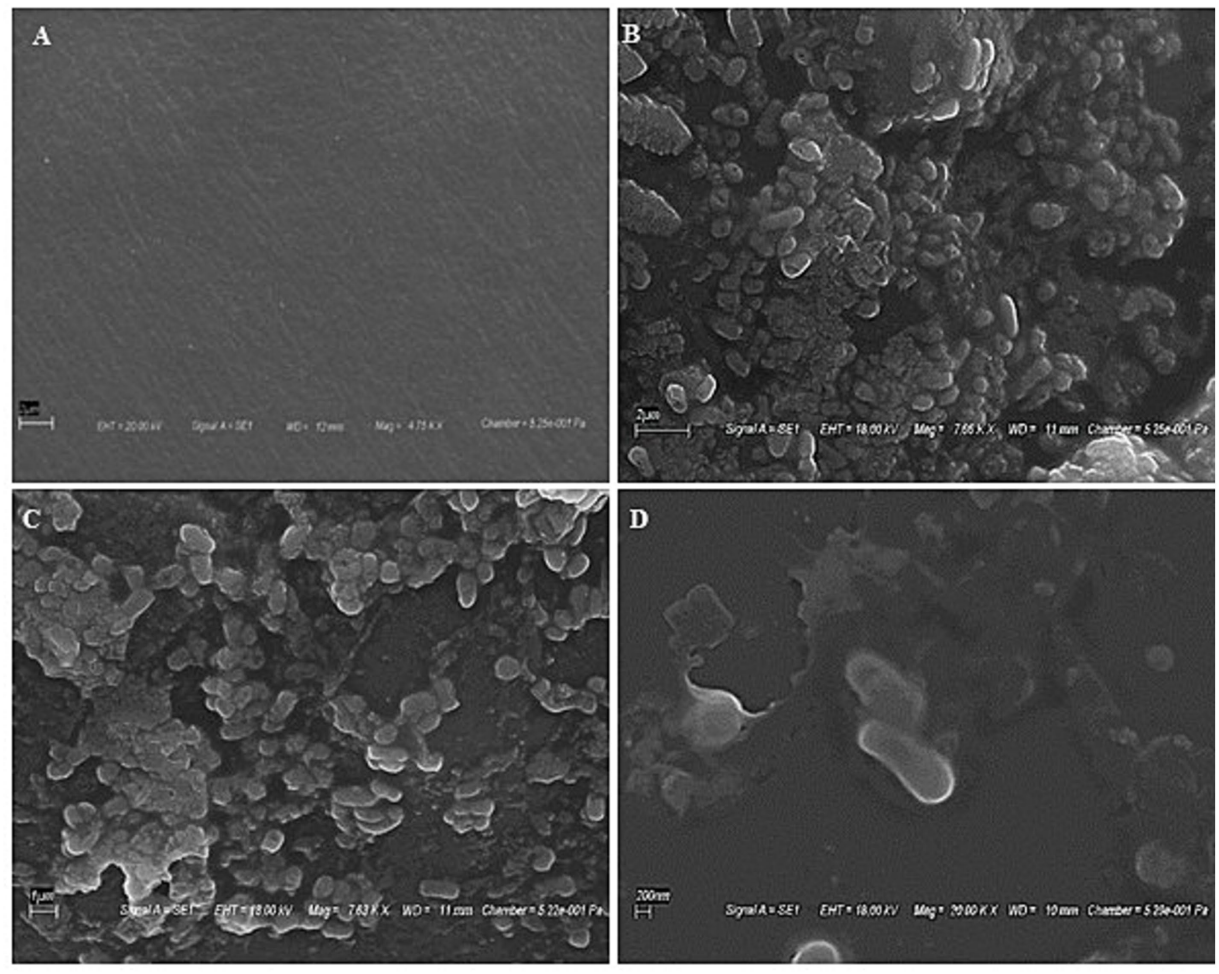
SEM images showing bacterial colonization over plastic films **(A)** Untreated film, **(B)** film at 2 μm, **(C)** film at 1 μm, and **(D)** film at 200 nm showing the presence of bacterial strain RS120 over the surface.

SEM scans recorded during biotreatment of PE film exhibited microbial colonization by strain RS120 on the surface, as shown in Figure 2. Microbial aggregates and dissemination were observed on the PE surface during the co-incubation. Conversely, the PE surface treated without strain showed no signs of aggregation and was used as a control for comparison (Figure 2A). As a result, the attachment of the bacterial strain *M. niabensis* RS120 was the cause of these highly apparent observed changes in visibility. The bacterium *B. niabensis*, also known as *M. niabensis*, produces surfactants that make plastic more accessible to microbial strains for colonization (Alemán-Vega et al., 2020). This can lead to a higher breakdown efficiency.

### 3.3 Characterization of PE degradation

#### 3.3.1 GC–MS analysis

**Figure 3:**
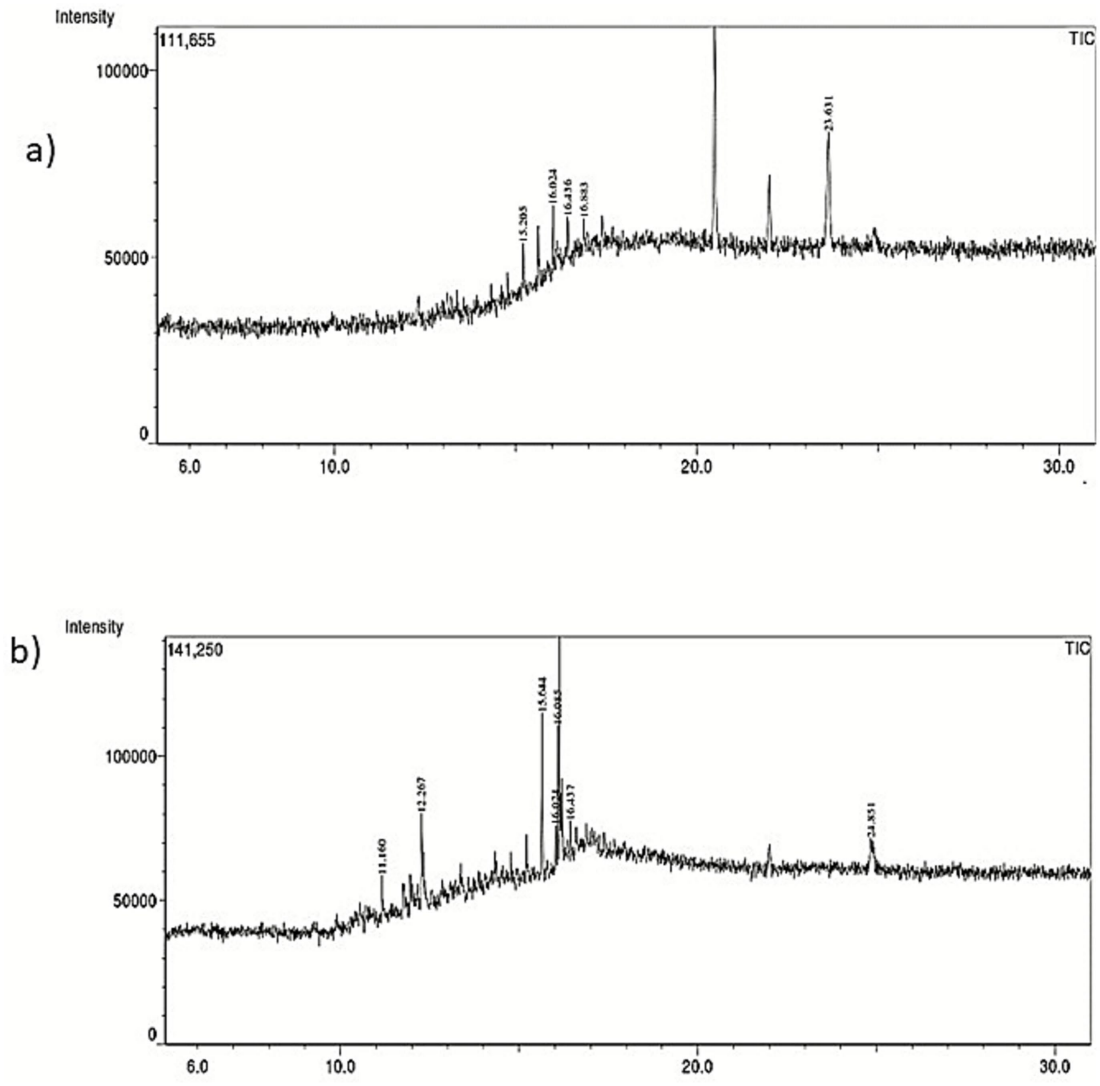
GC–MS chromatogram indicating chemical structure changes in **(a)** untreated and **(b)** treated with *M. niabensis* RS120.

The components of the shredded PE differed significantly from those of the untreated control, as determined by GC–MS analysis. Larger polymeric compounds nonaconate (C_29_H_60_) and tetra-tetracontane (C_44_H_90_) at retention times 15.62 and 16.02 were found in the untreated (Figure 3A). However, as a result of *M. niabensis* breaking down PE, the extract contained short chains of (1–2, propanediol, 3-phenyl (C_9_H_12_O_2_), and benzene, 1, 1′-(cyclobutanediayl) (C_16_H_16_) alkanes, carboxylic acids, and other compounds (listed in Table 2). Furthermore, several byproducts, including short-chain hydrocarbon compounds ranging from C_9_ to C_17_, were also found in the degraded PE extract; these may result from biodegradation.

**Table 2:** List of identified compounds detected in degraded and undegraded PE using GC–MS analysis.

Degraded MDPE		Un-degraded MDPE	
R* time	Compound detected	R time	Compound detected
11.10	Heptadecane (C17H36)	15.20	Eicosane (C20H42)
12.20	Benzene,1,1′- (cyclobutanediayl) (C16H14)	15.62	Tetratetracontane (C44H90)
15.64	1–2, propanediol, 3-phenyl (C9H12O2)	16.02	Tetratetraconate (C44H90)
16.02	Tetracosane (C24H50)	16.43	Nonaconate (C29H60)
16.08	(2,3-Diphenylcyclopropyl) methyl phenyl sulfoxide, trans-	16.88	Tetracosane (C24H50)
16.13	Tetracosane (C24H50)	20.49	--
16.17	(2,3-Diphenylcyclopropyl) methyl phenyl sulfoxide, trans-(C17H16)	22.00	--
16.20	(2,3-Diphenylcyclopropyl) methyl phenyl (C22H20)	23.60	1, 4-epoxynapthalene-1(2H) methanol
16.43	Tetracosane (C24H50) -Alkane		
24.85	2-tert-Butyl-4-6-bis (3-5-di-tert-butyl)		

*Retention time.

#### 3.3.2 FTIR study

**Figure 4:**
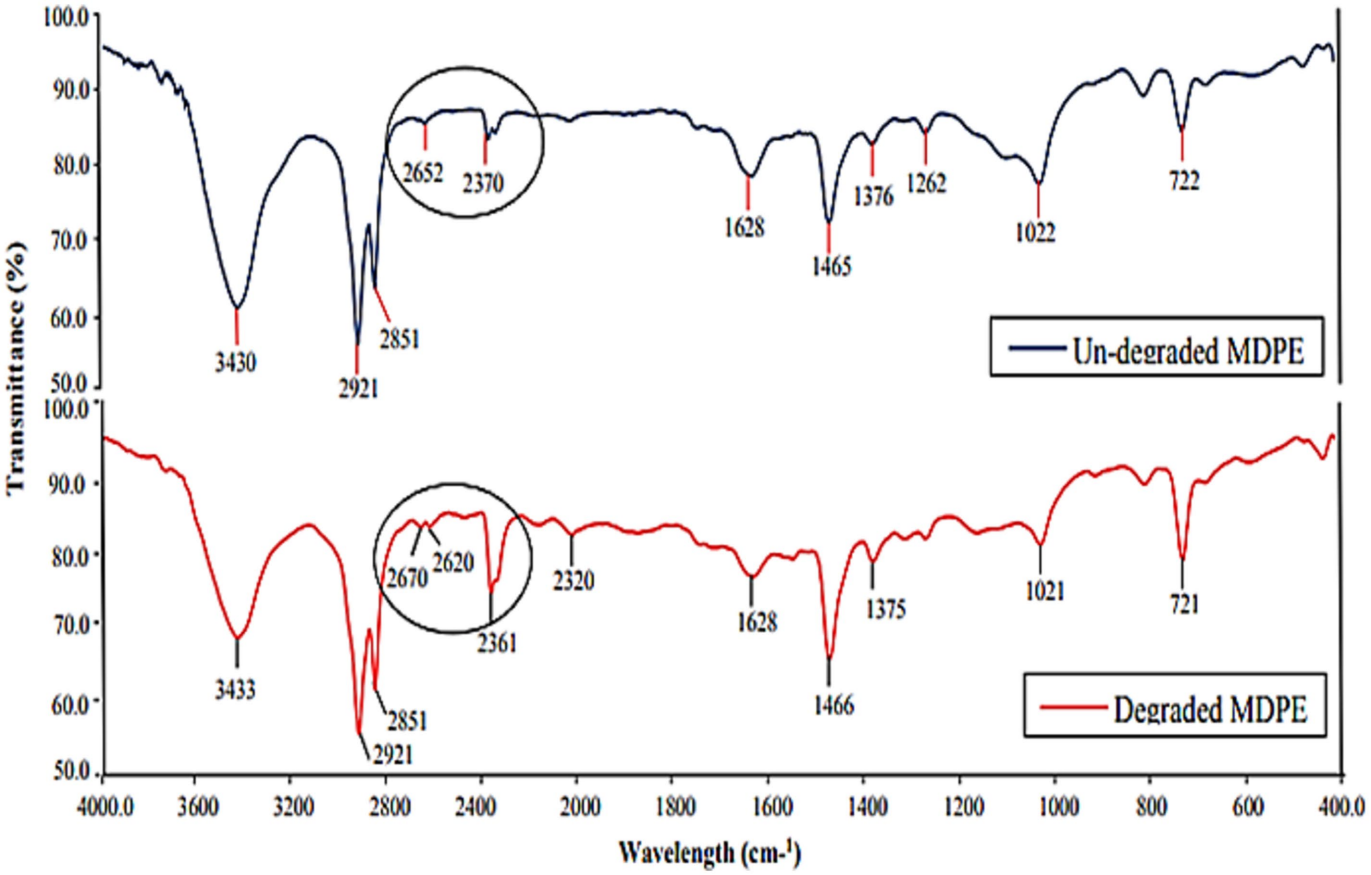
Infrared tests of the untreated and the treated group of PE after 30 days of treatment with strain *M. niabensis* RS120.

FT-IR analysis revealed alterations to the side chain and changes in functional groups induced by microbial enzymatic activity. According to Shah et al. (2007), the peaks at 2921 cm^−1^ and 2,851 cm^−1^ that are discovered to be common in both the degraded and undegraded film lie within the absorbance range of 2,800 to 3,000 cm^−1^, which is comparable to C–H stretching, the presence of alkanes, and the peak that is brought on by vibrations in the C=C bond stretching. Instead of a single peak at 2610 cm^−1^, the weakened PE film displayed two shouldered peaks at 2590 cm^−1^ and 2,610 cm^−1^ (Figure 4). In degraded PE, a peak at 2361 cm^−1^ showed longer and had a sharp, pointed appearance rather than being tiny in undegraded PE. The biotreatment of *M. niabensis* was thought to be the cause of these alterations in functional group and chemical shifts.

#### 3.3.3 XRD and DSC analysis

**Figure 5:**
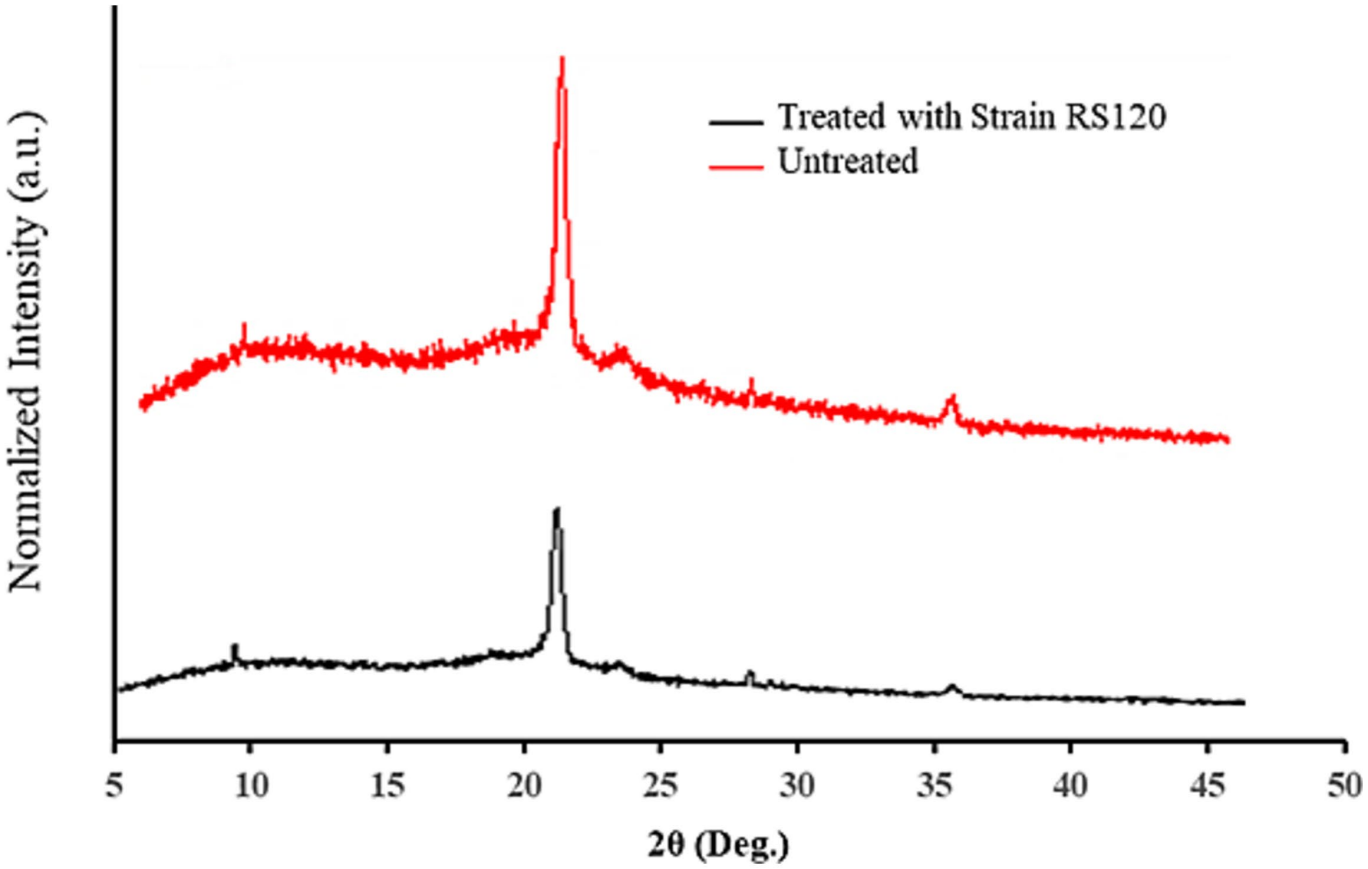
XRD spectra of untreated and treated PE with strain *M. niabensis* RS120.

XRD spectra showed a sharp, distinguished, large peak at 21.47 along with two minor peaks at 9.46 and 36.21 of the angular position 2θ in the undegraded PE film. Conversely, small shifts in the peak locations at 21.43 and 36.15 were observed in the degraded PE film, while the peak at 9.46 completely disappeared. The results specified that the intensity of the peaks illustrated in Table 3 and indicated as vertical coordinates in Figure 5 in degraded PE was significantly diminished after 30 days of incubation with *M. niabensis* in liquid mineral medium. The significant decrease in the relative height counts (Table 3) of the peaks indicated a decrease in the percent crystallinity (6.23%). Thus, it may be inferred that there may have been a change in the sample’s microstructure, including preferred orientation, crystallite size, and atomic arrangement in the films. These observed differences indicate a significant change in biodegradation caused by the *M. niabensis*.

**Table 3:** XRD analysis of PE.

Distribution name	Pos. [°2θ]	Height [cts]	FWHM Left [°2 θ]	d-spacing [Å]	Rel. Int. [%]
Before biotreatment	9.4620	155.66	0.1023	9.34717	15.04
	21.4750	**1034.82**	0.3326	4.13793	100.00
	36.2119	65.81	0.4093	2.48069	6.36
After biotreatment	21.4392	**790.29**	0.3326	4.14477	100.00
	36.1588	54.79	0.6140	2.48421	6.93

#Bold values indicate difference in height of peak in treated and untreated PE.

**Figure 6:**
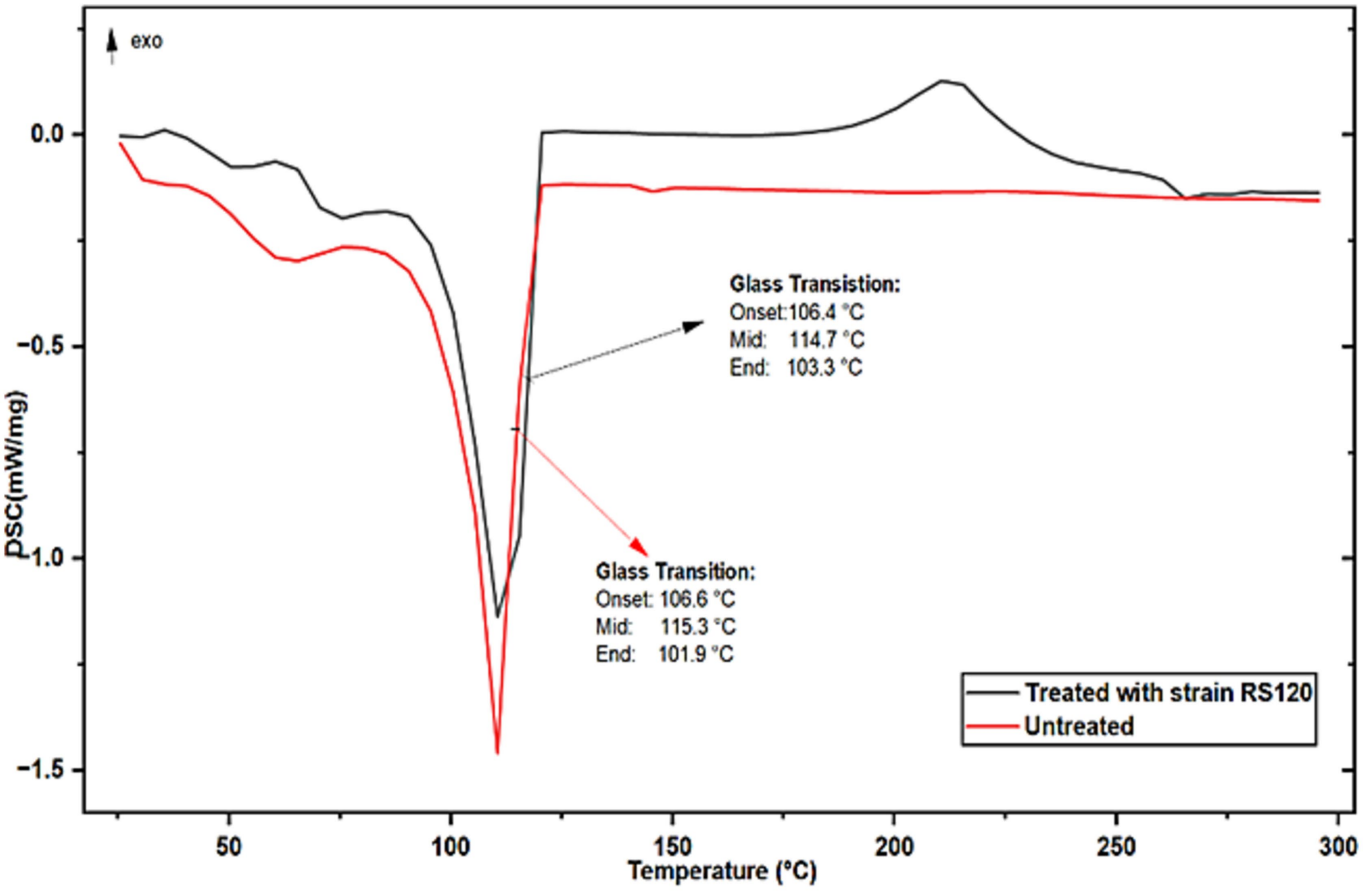
DSC spectra of untreated and treated PE with strain *M. niabensis* RS120.

The biologically treated and untreated PE strips were analyzed by DSC over a wide temperature range (200 °C to 300 °C) to determine their melting points. Results are shown in Figure 6, which represent the DSC curves of the thermally homogeneous material, as indicated by a single sharp exothermic melting peak at 114.70 °C. This value is somewhat lower than that of the control (T_m_ = 115.30 °C) PE. As reported in the literature, the cyanobacterial *Nostoc carneum* degrades low-density polyethylene (LDPE) and produces similar results (Sarmah and Rout, 2019). Both the melting temperature and the glass transition depend on the mobility of the polymer chains, which in turn depends on the chemical structure. Therefore, these properties can be used to identify a polymer with a specific chemical structure.

### 3.4 Determination of weight loss percent

Weight loss was recorded at 7 day intervals for a maximum of thirty days of biotreatment. The significant reduction in the total quantity of PE films demonstrates how effectively the marine bacteria biodegrade the incredibly strong polymer structure when the plastic serves as the sole carbon and energy source. Following the 7^th^, 14^th^, 21^th^, and 30^th^ days of treatment, the mean dry weight loss ± SD% was 0.78 ± 0.01, 1.58 ± 0.02, 2.45 ± 0.03, and 3.30 ± 0.03, correspondingly. The possibility that the bacteria took advantage of the carbon released from PE films during the incubation period is a reasonable explanation (Sardar, 2025). The observed weight decrease is thought to result from the oxidative fragmentation of the polymeric chain’s carbon backbone, generating volatile or gaseous species.

### 3.5 Enzyme activity

#### 3.5.1 Peroxidase and dehydrogenase

**Figure 7:**
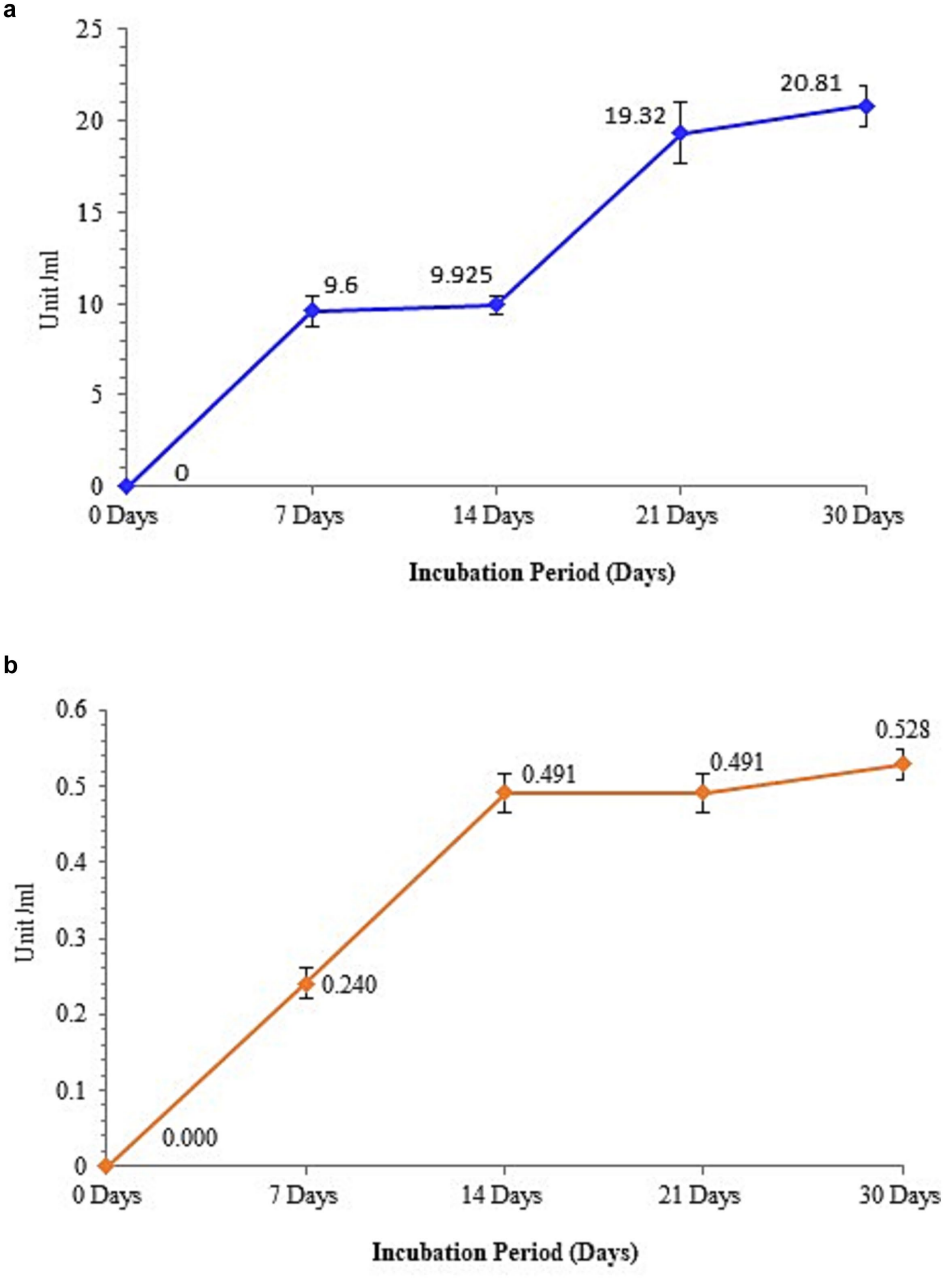
Enzyme assay showing **(a)** peroxidase activity and **(b)** dehydrogenase activity by strain RS120.

Peroxidase activity increased during the 7 and 21 day biotreatment periods, as shown in Figure 7a. However, comparatively sluggish increases were also observed at 14 and 30 days. Microbial oxidative enzymes, such as peroxidases, can catalyze the oxidative cleavage of carbon–carbon bonds in various organic molecules, thereby enhancing the biodegradation process. Consequently, the altered shape and form of PE with weight loss demonstrate the effectiveness of peroxidase in breakdown using M. niabensis. For the effective biodegradation of waste PE, using a strain that generates peroxidase is typically a feasible biotechnique. Moreover, it was verified that the dehydrogenase enzyme activity was high during the biotreatment until the first 14 days of incubation; at that point, it declined and then gradually increased (Figure 7b). According to Elumalai et al. (2021) and Abbasian et al. (2015), the enzyme dehydrogenase breaks down large hydrocarbon molecules into smaller ones.

## 4 Discussion

The rapid production of plastic waste in the environment has led to several environmental risks. The sea has an enormous reservoir of bioactive compounds that can be used to clean up contaminants from human activity. The conversion of TTC into TPF by marine bacteria, which is essential for their survival and growth during biodegradability tests of PE in the absence of carbon, confirmed this potential. Besides this, some marine bacteria screened for the biodegradation of HDPE include *Bacillus* spp. and *Pseudomonas* sp. (Devi et al., 2019), as well as *Pseudomonas* sp. and *Arthrobacter* sp. (Balasubramanian et al., 2010).

Total cellular fatty acid profiling of the PE-degrading strain RS120 discloses that the majorly branched fatty acids recorded are in agreement with the previously informed strain M. niabensis 4T19T of the same genus. The common major fatty acid components were C15:0 ante-iso, C16:0 iso, C17:0 ante-iso, C16:0, and C14:0 iso, as reported by Kwon et al. (2007).

The phylogenetic analysis also revealed proximity to *Metabacillus halosaccharovorans* C-1-0-8 and a distance from the outgroup, Ureibacillus thermosphaericus DSM 10633 T, among a dozen retrieved 16S gene sequence data sets for the genus *Metabacillus*. Although many marine-sourced microorganisms involved in plastic biodegradation have been reported earlier, M. niabensis is added to this list.

For a view of microbial attachment on the surface of PE, marine bacteria *Pseudomonas* sp. and *Lysinibacillus* sp. (Oliveira et al., 2021) and *Micrococcus flavus* (Sardar, 2025), strain RS120, also showed a similar strategy. Microbial cell attachment to the film surface may be driven by the secretion of substances with maximum early activity, such as sugars, proteins, enzymes, and organic acids. For the initiation of biodegradation, biofilm formation is observed as a key factor. Plastic’s hydrophobicity, roughness, topography, and electrostatic interactions all contribute to its adsorption, desorption, and deterioration.

GC–MS analysis reveals the emergence of low molecular n-alkane compounds like heptadecane (C_17_H_36_) and tetracosane (C_24_H_50_) with benzene and phenol-containing intermediate products in the weathered PE shown in Figure 3. A recent study by Liu et al. (2022) described the degradation of LDPE films in 3 months by *Bacillus velezensis* and also showed the intermediate product tetracosane (C_24_H_50_) among C_24_–C_29_ n-alkanes, which corroborates my findings. The presence of these by-products after PE biodegradation and their detection provide important evidence that PE undergoes depolymerization. Moreover, the creation of alkane compounds such as eicosane, tetratriacontane, nonaconate, and tetracosane in degraded PE (Tiago et al., 2023) explicated the direction of the breakage of the polymer chain, originating carbonyl radicals that can react with an alkoxy radical (R–O) on the PE chain (Norrish I-type reaction). According to a previous report by Awasthi et al. (2017), *Rhizopus oryzae*’s breakdown of LDPE releases alkanes and carboxylic acids. Furthermore, another study reported that GC–MS analysis of PE showed the production of fatty acids due to enzymatic activity. The degradation of plastic is also attributed to extracellular enzymes produced by microorganisms, such as laccase, lignin peroxidase, and esterase. According to a prior study, there are three stages in the biodegradation of PE. Initially, PE is degraded into oligomers, dimers, or monomers through biological oxidation or enzyme-mediated processes. Due to further oxidation reactions, the polymer chains are broken down, forming fatty acids. Finally, fatty acids are metabolized by microorganisms into the small molecules CO₂ and H₂O (Gao et al., 2024).

FTIR reveals structural destabilization and changes in the chemical structure of plastic treated with *M. niabensis*, precisely depicting the biodegradation of PE. This study demonstrates that shifts and changes in peak transmittance, as well as a decrease in side-chain peak and functional group transmittance, were achieved due to the enzymatic degradation of PE. A similar result was also depicted by *Bacillus weihenstephanensis*. The shift from wave number 1634 to 1,629 cm−^1^ in the treated film shown in Figure 4 corresponds to the carbonyl group, which is related to oxidation of the PE sample (Rajandas et al., 2012). At the same time, these peaks suggest that the metabolic activity of these strains destroys the chemical inertness of PE by inducing unstable and reactive bonds with hydroxyl groups (Matsunaga and Whitney, 2000; Ng et al., 2018). Furthermore, as outlined by Sen and Raut (2015), some extracellular enzymes can introduce oxygen atoms into the chemical structure of PE, suggesting that strain RS120 may also exhibit a similar hydrolase activity. Therefore, GC–MS and FTIR analysis revealed the emergence of a novel molecule and a modified peak in degraded PE, which may be attributed to the activity of enzymes released under bio-stress conditions.

XRD can be used to evaluate the degree of crystallinity of the polymers. PE’s orthorhombic crystal structure is characterized by reflections at 2θ peaks at 21.43° and another peak created at 2θ = 36.15° (Morancho et al., 2006). Consequently, biodegradation processes that shrink the crystallites and ultimately expand the amorphous region decrease crystallinity. Balasubramanian et al. (2010) reported a comparable decrease in crystallinity following incubation with bacterial strains. According to the reported data, bacterial enzymes may penetrate and disrupt crystalline regions, leading to a loss of chain order due to the disordering of crystalline domains, which results in an overall decrease in crystallinity. A further drop in crystallinity occurs due to the attack on smaller crystals. A similar observation was also noted by Wu et al. (2023) in the biodegradation of PE by *Bacillus paramycoides.*

The observed melting peak of shredded PE (T_m_ = 114 °C) and the shift in unshredded PE (T_m_ = 115 °C) may be due to the breakdown of branches and changes in PE density. According to Menczel and Prime (2008), the characteristic phase transition temperature can be influenced by the magnitude of polymer branching, as well as by additives, contaminants, and particle size. According to published research, high-density PE exhibits somewhat larger melting peak temperatures due to its greater degree of branching (Camacho and Karlsson, 2001; Sorolla-Rosario et al., 2022). Since the complex, mixed branching structure of LDPE molecules influences their crystallization during the cooling phase of the DSC experiment, resulting in a wide range of lamellar thicknesses, these results make sense. The density of the polymer also affects its correlation with the melting temperature of PE; higher-density PE exhibits a higher melting temperature, and vice versa.

The degradation efficiency can be calculated by measuring the weight loss of PE films (Yang et al., 2020). Here, the weight loss rate of the PE film gradually increased with increasing co-culture time. A different study by Sudhakar et al. (2008) found that after a prolonged incubation, *B. sphericus* caused a dry weight loss of approximately 3.5%, while *B. cereus* caused a dry weight loss of around 2% in treated PE films. The observed weight loss is close to my findings. Moreover, *Exiguobacterium* and *B. subtilis* colonize and degrade PE by 4.7 and 12.1%, respectively (Muthukumar et al., 2014). The deteriorating surface area resulting from direct contact of bacterial strains with the polymer and subsequent consumption was linked to the weight loss percentage of DPE. Low molecular weight chains in PE are consumed, and chain scission arises at the end or branch of the polymer chain rather than the center of the molecule. Such disintegration of PE releases free carbon from HDPE sheets throughout the incubation in the liquid broth, resulting in a weight reduction. However, the long hydrophobic polymer chains cannot be transported directly into microorganisms via outer membranes.

A hydrolytic breakdown of the polymer is triggered by the release of extracellular enzymes that attach to it (Wilkes and Aristilde, 2017; Sheel and Pant, 2018). As previously noted, the periplasmic dehydrogenase from Gram-negative bacteria plays a crucial role in the biodegradation of polyvinyl alcohol (PVA) (Kawai and Hu, 2009). Periplasmic PVA dehydrogenase, an active enzyme that hydrolyzes PVA, was shown to be present in *Sphingopyxis* sp. 113P3. To obtain energy, microorganisms speed up chemical reactions that release electrons from polymers. Among bacterial oxidative enzymes, peroxidase exhibited the capacity to catalyze the oxidative cleavage of carbon–carbon bonds, hence increasing the rate of biodegradation (Mohammadi et al., 2022). Hydroquinone peroxidase, the first microbial enzyme identified as being involved in the breakdown of polystyrene, was isolated from Azotobacter beijerinckii HM121 (Oyewole et al., 2022). Discovering bacteria and enzymes that can break down plastic polymers opens the door to bioremediation, an environmentally beneficial, natural way to reduce pollution. In addition to minimizing damage to marine life and restoring marine biodiversity, it also helps to prevent the accumulation of microplastics in marine ecosystems. It converts plastics into biomass, carbon dioxide, and water.

**Figure 8:**
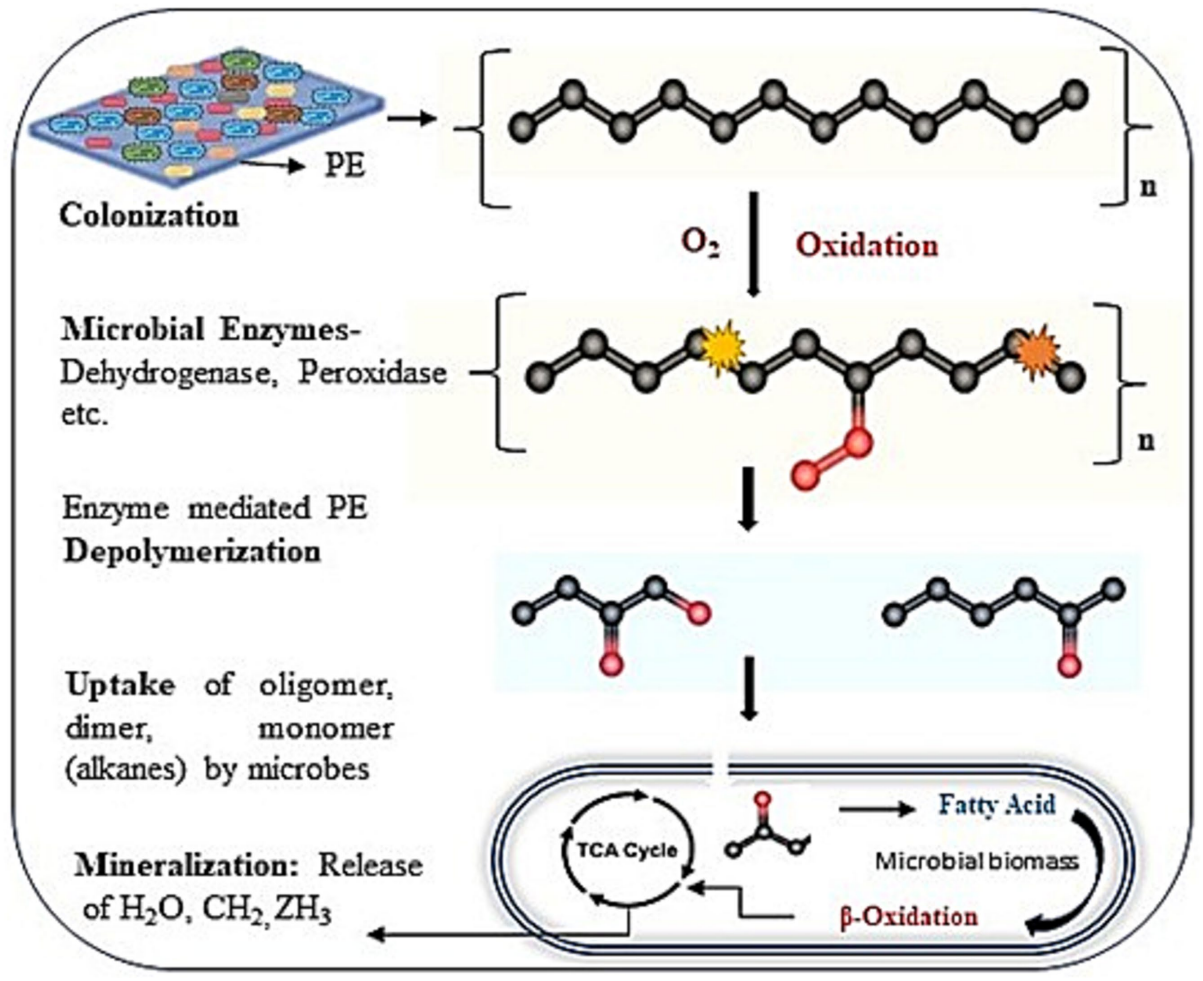
A projected mechanism of microbial enzyme-mediated PE biodegradation.

Here, Figure 8 illustrates a proposed mechanism for the biodegradation of PE through microbial attack. The four steps of PE biodegradation, including colonization, depolymerization, assimilation, and mineralization, are described (Restrepo-Flórez et al., 2014; Ali et al., 2021). Microbial aggregates, composed of cells and extracellular polymers, can adhere to plastic surfaces or to one another during the colonization phase, forming a complex biofilm that is visualized in Figure 2 (Flemming et al., 2016). The polymer surface’s chemical inertness is decreased, and its hydrophobicity is compromised as a result of microorganisms interacting with different extracellular enzymes (peroxidase, hydrolases) generated by microbial strains (Gaur et al., 2022). After that, several enzymes released by the microbial strain attack the plastic’s molecular chain, breaking the hydrolysable chemical bond or the terminus of the chain (Othman et al., 2021; Show et al., 2021). As a result, monomers and dimers would develop, lowering the plastic’s molecular weight (600 Da) (Santacruz-Juarez et al., 2021; Santo et al., 2013; Spina et al., 2021). Oxygen-containing functional groups, mentioning hydroxyl and carbonyl groups, were formed concurrently by further oxidation under the influence of oxygen. The generated oligomers and broken molecular chains are discharged into the environment. The microbial enzyme system (such as oxidoreductase, esterase, and lipase) can more readily identify and target the degradation intermediates because some of the oligomers with lower molecular weights can penetrate the strain’s cell membrane and enter the microbe (Shah et al., 2008). Considering the chemical similarity between PE and alkanes, it is believed that the metabolic pathways of alkanes and polymer breakdown are quite comparable when the sizes of polymer molecules are reduced to an appropriate range for enzyme attack (Jeon and Kim, 2015). According to Van Beilen et al. (2003), terminal oxidase later catalyzes the breakdown of PE intermediates to produce alcohols, which are then further oxidized by alcohol and aldehyde dehydrogenase, and the resultant fatty acids enter the oxidation cycle. Second, subterminal oxidation monooxygenase catalyzes the breakdown of PE intermediates to form secondary alcohols, which are then oxidized to ketones by alcohol dehydrogenase. This is followed by *β*-oxidation, which turns oxidized carboxylic molecules with an even number of carbon atoms into either propionyl CoA or acetyl CoA (if the number of carbons is odd). Propionyl-CoA carboxylase is responsible for carboxylating propionyl-CoA into succinyl-CoA (Gravouil et al., 2017). The tricarboxylic acid (TCA) cycle is entered by acetyl-CoA and succinyl-CoA. The reducing power (NADH and CoQ_10_H_2_) produced by this cycle is utilized in the respiratory chain to generate ATP, which is required for the production of new microbial biomass through replication mechanisms. Certain short-chain, water-soluble intermediates created by depolymerization are detected by receptors during absorption and subsequently transferred to microorganisms across the membrane, where they participate in a range of metabolic processes and stimulate cell division. Ultimately, a few metabolites and unassimilated products were fully absorbed, utilized, and transformed into energy, carbon sources, carbon dioxide, and water molecules during the mineralization process.

## 5 Conclusion

Microbes from marine sources can bioremediate untreated PE, as this study shows. Prolonged incubation caused by the newly found *M. niabensis* of 22 known species of the genus, which adhere to the surface as a biofilm, further altered the PE structure and shifted peak absorbance. This resulted in the creation of a novel intermediate by-product, which was examined using various analytical tools. The biotreatment method is an environmentally beneficial approach to removing plastic from the environment and converting it into biomass in a laboratory setting. These findings demonstrated the potential of marine bacteria in waste management, and future studies will focus on tackling plastic waste, promoting a circular economy, and optimizing microbial populations for various plastics and practical applications.

## Data Availability

The data presented in this study are publicly available. The data can be found at: https://www.ncbi.nlm.nih.gov/genbank, accession PP858188.

## Supplementary material

SUPPLEMENTARY FIGURE S1 Screening of PE-degrading bacteria using Bushnell and Haas broth (minimal media) showed **(a)** MDPE film with the RS120 strain, **(b)** MDPE film only, and **(c)** Strain RS120 without MDPE.

SUPPLEMENTARY FIGURE S2 GC-MS Chromatogram of total cellular fatty acids of *M. niabensis* strain RS120.
