# Supplementary figures and images for: Discovery and biodegradation characterization of polyethylene by Metabacillus niabensis
Source: Front Microbiol. 2025 Dec 3;16:1693690. doi: 10.3389/fmicb.2025.1693690 (PMC12709022; doi:10.3389/fmicb.2025.1693690)

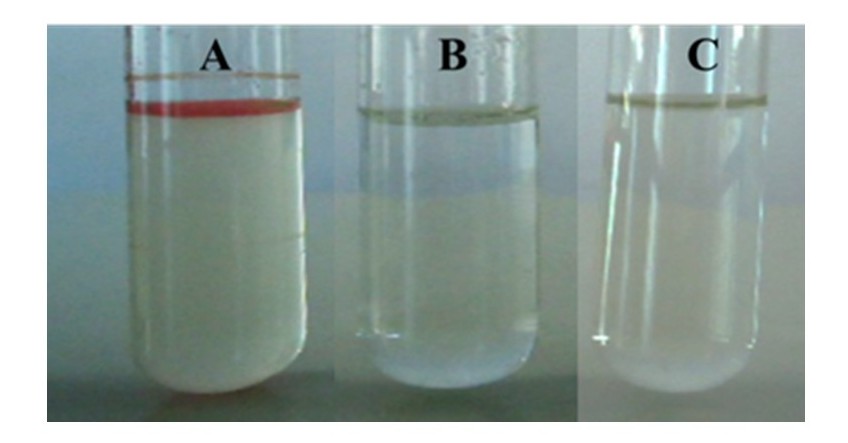

Supplement: SUPPLEMENTARY FIGURE S1 — Screening of PE-degrading bacteria using Bushnell and Haas broth (minimal media) showed (a) MDPE film with the RS120 strain, (b) MDPE film only, and (c) Strain RS120 without MDPE. [file Image_1.jpeg]

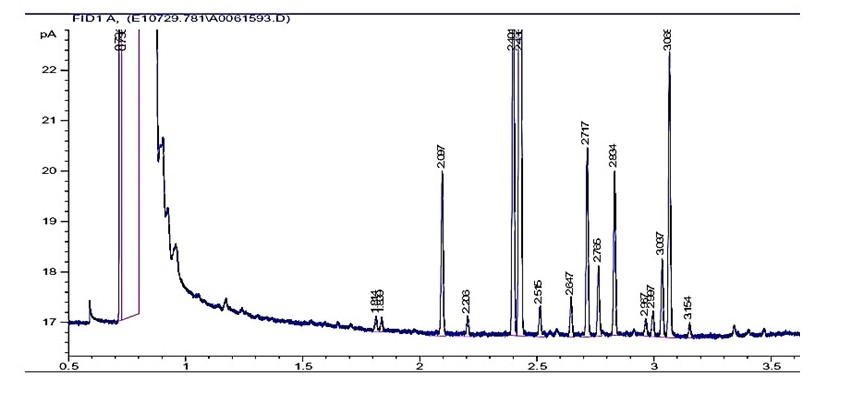

Supplement: SUPPLEMENTARY FIGURE S2 — GC-MS Chromatogram of total cellular fatty acids of M. niabensis strain RS120. [file Image_2.jpeg]
